# Modeling SF-6D Health Utilities: Is Bayesian Approach Appropriate?

**DOI:** 10.3390/ijerph18168409

**Published:** 2021-08-09

**Authors:** Samer A. Kharroubi

**Affiliations:** 1 Department of Nutrition and Food Sciences, Faculty of Agricultural and Food Sciences, American University of Beirut, P.O. Box 11-0236, Riad El Solh, Beirut 1107-2020, Lebanon; sk157@aub.edu.lb; Tel.: +961-1-350-000 (ext. 4541); 2 School of Health and Related Research, The University of Sheffield, Regent Court, 30 Regent Street, Sheffield S1 4DA, UK

**Keywords:** nonparametric Bayesian methods, preference-based health measures, SF-6D system, standard gamble

## Abstract

Background: Valuation studies of preference-based health measures like SF6D have been conducted in many countries. However, the cost of conducting such studies in countries with small populations or low- and middle-income countries (LMICs) can be prohibitive. There is potential to use results from readily available countries’ valuations to produce better valuation estimates. Methods: Data from Lebanon and UK SF-6D value sets were analyzed, where values for 49 and 249 health states were extracted from samples of Lebanon and UK populations, respectively, using standard gamble techniques. A nonparametric Bayesian model was used to estimate a Lebanon value set using the UK data as informative priors. The resulting estimates were then compared to a Lebanon value set obtained using Lebanon data by itself via various prediction criterions. Results: The findings permit the UK evidence to contribute potential prior information to the Lebanon analysis by producing more precise valuation estimates than analyzing Lebanon data only under all criterions used. Conclusions: The positive findings suggest that existing valuation studies can be merged with a small valuation set in another country to produce value sets, thereby making own country value sets more attainable for LMICs.

## 1. Introduction

Currently, there are a number of preference-based measures of health-related quality of life (HRQoL) available. Some of these measures include the EuroQol five-dimensional (EQ-5D) questionnaire [1], Healthy Utilities Index 2 (HUI2) and HUI3 [2,3], Assessment of quality of life (AQoL) [4], Quality of Well-Being scale (QWB) [5], and the six-dimensional health state short form (SF-6D) [6], in addition to a growing set of condition-specific measures [7,8]. All of these measures provide empirically derived health state utility values that can be used to calculate quality-adjusted life years (QALYs), a commonly used effectiveness measure in a specific form of cost-effectiveness analysis (CEA): cost-utility analysis (CUA). CUA involves comparison of the costs of a treatment with its effectiveness expressed in units, such as QALYs, gained for use in CEA [9].

The SF-6D has become one of the most widely adopted HRQoL measures, primarily in the United Kingdom (UK) [6]. It has also achieved extensive usage internationally in different countries across the globe, reaching China [10], Japan [11], Hong Kong [12], Brazil [13], Portugal [14], and Australia [15]. Further, it is largely available for use in datasets since it is derived from the original short form 36 health survey (SF-36) [16]. In the Middle East, conducting valuation studies is a relatively new research area, with only a single pilot valuation study investigating the feasibility and acceptability of adopting the standard gamble (SG) technique to derive value sets for the Arabic version of SF-6D in Lebanon [17].

For countries with small populations or low- and middle-income countries (LMICs), such as Lebanon, the cost of conducting valuation studies to derive country-specific value sets can be prohibitive, especially in the context of data collection post the COVID-19 pandemic. For instance, collecting valuation data by face-to-face interviews could be expensive and most often time consuming, and the number of such interviews would be relatively small (e.g., only 126 interviews for the Lebanon pilot study). It could be argued that valuation studies may be conducted online, thereby making the data collection cheaper and quicker. However, this may not be achievable for every country. For some LMICs, conducting an online survey may not be feasible and obtaining a representative sample of the general population based on sociodemographic characteristics may not be achievable. This means that other countries’ value sets, like UK or US values, could be used instead to derive QALYs. However, these value sets may not be representative of the country’s own population, which, in turn, could potentially impact on the validity of the resource allocation decisions made.

Advancement in statistical modeling, such as Bayesian inference methods [18], facilitates the use of the results of one country to improve those in another country by using the results in one country as informative priors. As a result, the second country’s generated utility estimates will be more precise than analyzing its data individually. Therefore, the use of additional evidence from country 1 may allow a reduction in the sample size in country 2, thereby achieving similar accuracy as that obtained with a full-sized valuation study in that country [19]. This sort of analysis tends to be hugely important in countries without the same capacity to perform large-scale evaluation exercises, particularly for those with smaller populations or LMICs. In Kharroubi [20], a nonparametric Bayesian model was developed to allow results from one country to be used as prior information for a study in another. This model was applied for the analysis of a US EQ-5D valuation study using the already existing UK data [21]. Recently, this model was also applied for SF-6D HK and Japan alongside the existing UK data, respectively [22,23].

The aim of the present study was to explore if such an approach could be used in countries with small populations and various demographic compositions, work, cultures, and languages, and if so, how generalizable these approaches may be by using experiences from a European country to facilitate the analysis of a value set in another Asian country or LMIC. This was investigated using a case study for SF-6D Lebanon and UK datasets, where a sample of health states valued in the Lebanon study were analyzed alongside the existing UK dataset, and the resulting estimates were then compared to those generated modelling the Lebanon dataset on its own. Despite the fact that this paper is not proposing new methodological advances, given the model presented here is a replica of that used in the Kharroubi et al. [20,21,22,23] articles, it further emphasizes the key points made in these studies, such as the better performance of the nonparametric Bayesian approach in producing precise estimates.

First, the UK and Lebanon SF-6D valuation surveys in addition to the datasets are summarized. The Bayesian methodology is outlined second whereas the findings are set out third. To this end, the findings are discussed in addition to some limitations and suggestions of possible future outlooks.

## 2. Materials and Methods

### 2.1. The SF-6D

The SF-6D is a generic health state measure defined by six multi-level dimensions of health: physical functioning, role constraints, social functioning, pain, mental health, and vitality [6]. Each dimension is assigned between 4 and 6 levels. An SF-6D health state is described by a six-digit number generated by selecting a level from each dimension, starting with physical functioning and ending with vitality. Thus, state 111,111 denotes the full health state and state 645,655 denotes the worst health state, referred to as “the pits”. Different combinations generate 18,000 possible health states

### 2.2. The Valuation Survey and Data Set

#### 2.2.1. UK

A total of 836 respondents from the general public in the UK valued 249 SF-6D health states. The selection of respondents along with the selection of health states are provided elsewhere [6]. Using the McMaster “ping pong” form of the SG technique, each respondent was asked to rank and then value six of these states. Respondents were asked to value five of the six SF-6D health states against the full health and the “pits” using the SG approach. In the sixth SG question, respondents were asked to value the “pits”. Each respondent was given the option of choosing between (A) the certain prospect of being in the “pits” state and the uncertain prospect of full health or immediate death, or (B) the certain prospect of death and the uncertain prospect of full health or the “pits” state, based on their assessment of the “pits”. The probability of the best possible outcome occurrence was changed until the respondent was undecided between the two certain and uncertain outcomes. Every negative value was restricted to a value of −1, indicating that it was worse than death [24]. The rest of the health states were converted to a [0, 1] scale, with 0 and 1 indicating death and the full health state, respectively, using the following equation:

SGADJ = SG + (1 − SG) ∗ *P*,

where *P* represents the “pits” state valuation. Note that the utility scores derived from the SG technique take values between 0 and 1 except for those who had a “worse than death” evaluation. Note also that the values of SGADJ constitute the dependent variable (*y*) in the model outlined below.

Out of the original 836 respondents, 225 respondents were removed from the analysis for different reasons. For instance, 130 respondents proved to be ineligible because they failed to value the “pits” state, thereby generating an SGADJ value that was not possible. Out of the remaining 611 individuals, 148 values from 117 respondents were missing. This resulted in 3518 observed SG values for the 249 SF-6D UK health states. A more detailed explanation on this study is provided in Brazier et al. [6].

#### 2.2.2. Lebanon

The Lebanon valuation study included a sample of 49 SF-6D health states. These health states were selected and valued according to the UK selection and valuation procedures [16]. The interview approach was also based on that used in the UK study, though every respondent was asked to rank and then value eight different SF-6D health states.

Out of the 170 respondents contacted for an interview, 44 were excluded from the analysis based on the same exclusion criteria as the UK study [6]. Each of the remaining 126 respondents completed 8 SG values, resulting in a total of 1008 valuations. Among these, 16 health state values were missing. This resulted in 992 observed SG values over 49 SF-6D states. A further detailed description for each of these 49 health states is presented in Kharroubi et al. [17].

### 2.3. Modelling

In Kharroubi [20], a nonparametric Bayesian model was developed to allow results from one country to be used as prior information for a study in a new country. Using the full US and UK datasets, this model was applied to the analysis of the US EQ-5D valuation study alongside the existing UK data [21]. Recently, this model was also applied for SF-6D HK and Japan alongside the existing UK data, respectively [22,23]. Here, we aimed to explore whether the use of Lebanon valuations alongside the existing evidence from the UK produces better valuation estimates than analyzing the Lebanon data alone. The resulting estimates are then compared to those obtained using Lebanon data by itself (which we shall refer to henceforth as the crude model) via various prediction criterions, incorporating estimated against observed mean utilities, mean predicted error as well as root mean square error (RMSE).

Kharroubi [20] employs the model below:

(1) $$y_{ij}=1-\left\{\alpha_{j}(1-u\left(x_{ij}\right)\right\}+\varepsilon_{ij}$$

where for $i=1,2,\ldots ,I_{j}$ and j=1,2,…,J, $x_{ij}$ denotes the *i*th health state valued by respondent *j* in the Lebanon valuation and $y_{ij}$ represents the respondent *j*’s SGADJ value for that health state. The model also includes two random terms: an individual effect term $\alpha_{j}$ of respondent *j* and the error term $\varepsilon_{ij}$. Let $t_{j}$ be a vector of covariates for respondent *j*, such as age and gender or socio-economic factors of respondents, to which the following distributions have been assigned by Kharroubi [20]:

(2) $$\alpha_{j}∼LN\left(t_{j}^{T}\gamma ,\tau^{2}\right)\;\mathrm{and}\;\varepsilon_{ij}∼N\left(0,v^{2}\right),$$

where *γ* is the vector of coefficients for the covariates and $\tau^{2}$ and $v^{2}$ are further variance parameters to be estimated. That is, the distribution of the respondent effect $\alpha_{j}$ is then independent log-normal, resulting in a skewness that is also typically observed in valuation data, and $\varepsilon_{ij}$ are independent normally distributed errors.

Now assume u(x) and $u_{UK}\left(x\right)$ are the utility functions of health state **x** evaluated, respectively, in the Lebanon and the UK analyses. Kharroubi [20] formally assumed a multivariate normal distribution for the prior distribution of u(x) with mean:

(3) $$E\left(u\left(x\right)\right)=E\left(u_{UK}\left(x\right)\right)+\gamma +\beta^{T}x$$

and variance–covariance matrix:

(4) $$\mathrm{cov}\left(u_{UK}\left(x\right),u_{UK}\left(x^{\prime }\right)\right)+\sigma^{2}c\left(x,x^{\prime }\right)$$

where $E\left(u_{UK}\left(x\right)\right)$ represents the mean utility value for state **x** and $\mathrm{cov}\left(u_{UK}\left(x\right),u_{UK}\left(x^{\prime }\right)\right)$ denotes the variance–covariance matrix between and $u_{UK}\left(x^{\prime }\right)$ for two distinct health states **x** and **x**′ in the UK study. Note that both $E\left(u_{UK}\left(x\right)\right)$ and $\mathrm{cov}\left(u_{UK}\left(x\right),u_{UK}\left(x^{\prime }\right)\right)$ in Equations (3) and (4) are derived from analyzing the readily available UK data. We shall discuss the interpretation of these in more detail when considering the crude model later in this section. Note also that the use of $E\left(u_{UK}\left(x\right)\right)$ and $\mathrm{cov}\left(u_{UK}\left(x\right),u_{UK}\left(x^{\prime }\right)\right)$ offers the potential for using the results in the UK data as informative prior to the Lebanon utility function u(x). See Kharroubi [20] for a detailed description of this.

Consider Equations (3) and (4), note that health state $x=\left(x_{1},x_{2},\ldots ,x_{6}\right)$ represents a vector consisting of discrete levels on every health dimension of the SF-6D and γ, β, and $\sigma^{2}$ are unknown coefficients. Note also that the regression parameters γ and β represent, respectively, the intercept term and the slopes as each of the six dimensions (physical functioning, role constraints, social functioning, pain, mental health, and vitality) increases, whereas the term *c*(**x**, **x**′), defined below represents the correlation between the utilities u(x) and $u\left(x^{\prime }\right)$ for two distinct health states **x** and **x**′ in the Lebanon study. The interpretation of (3) is that the mean function represents a belief that the utility is more likely to be linear and additive in the different dimensions. Furthermore, the true function is set to fluctuate freely around this mean, conforming to its multivariate normal distribution, and so it may take any form at all. Additionally, if **x** and **x**′ are somehow similar, then the utilities u(x) and $u\left(x^{\prime }\right)$ will have high correlation *c*(**x**, **x**′), defined as follows:

(5) $$c\left(x,x^{\prime }\right)=\exp\left\{-\sum^{\;}b_{d}{\left(x_{d}-{x^{\prime }}_{d}\right)}^{2}\right\}$$

where for d=1,2,…,6, $x_{d}$ and ${x^{\prime }}_{d}$ represent the levels of dimension *d* in **x** and **x**′, respectively, and $b_{d}$ represents a roughness parameter, which controls how closely the actual utility function is to a linear form in dimension *d*. Details pertaining to this specific point can be found in Kharroubi [20].

The Bayesian model is finalized by assigning prior distributions to the (hyper)parameters *γ*, $\tau^{2}$, $v^{2}$, β, and $\sigma^{2}$. In the case when no specific prior information is available, it is appropriate to assign conventional noninformative prior distributions for all these parameters. Formally, we assume:

(6) $$p(\gamma ,\;\tau^{2},\;v^{2},,\;\sigma^{2})\propto \tau^{-2}v^{-2}\sigma^{-1}$$

Note that a uniform prior for *σ* was used, hence *p*$\left(\sigma^{2}\right)\propto \;\sigma^{-1}$ [25]. Note also that no distributions were given to the $b_{d}$ s. Generally, inference about these roughness hyperparameters in Gaussian models is problematic, and so it is preferred to set fixed values for them [18]. A discussion of one way to do this is considered in Section 3.

To this end, it follows from Kharroubi et al. [20] that the mean health state utility in model (1) is:

$$\bar{u}\left(x\right)=1-\bar{\alpha }\left\{1-u\left(x\right)\right\}$$

where $\bar{\alpha }$ denotes the expected value of α over the entire population. Note that, if $\bar{\alpha }=1$, then $\bar{u}\left(x\right)=u\left(x\right)$, which is not generally the case. Kharroubi et al. [18] provide more explanation on the computation of $\bar{\alpha }$.

A final note regarding the specification of the crude model and the distributions placed on the model parameters: in brief, the crude model is defined analogously to model (1) i.e.,

$$y_{ij}=1-\left\{\alpha_{UK_{j}}(1-u_{UK}\left(x_{ij}\right)\right\}+\varepsilon_{ij}$$

where $x_{ij}$ is the *i*th health state valued by respondent *j* in the UK valuation and $y_{ij}$ is the UK respondent *j*’s SGADJ value for that health state, $\alpha_{UK_{j}}$ is an individual effect term of UK respondent *j*, and $\varepsilon_{ij}$ is a zero mean random error term. Note that the distributions of $\alpha_{UK_{j}}$ and $\varepsilon_{ij}$ are defined analogously to those in Equation (2). Furthermore, Kharroubi et al. [18] assumed a multivariate normal distribution for the prior distribution of $u_{UK}\left(x\right)$ with a mean:

$$E\left(u_{UK}\left(x\right)\right)=\gamma_{UK}+\beta_{UK}{}^{T}x$$

and variance–covariance matrix:

$$\mathrm{cov}\left(u_{UK}\left(x\right),u_{UK}\left(x^{\prime }\right)\right)=\sigma_{UK}{}^{2}c_{UK}\left(x,x^{\prime }\right)$$

where $\gamma_{UK},\;\beta_{UK}$, and $\sigma_{UK}{}^{2}$ are unknown parameters and $c_{UK}\left(x,x^{\prime }\right)$ is the correlation between $u_{UK}\left(x\right)$ and $u_{UK}\left(x^{\prime }\right)$ for two distinct health states **x** and **x**′ in the UK study, defined analogously to Equation (5). The crude model is completed by assigning conventional noninformative prior distributions to all these (hyper)parameters. See Kharroubi et al. [18] for a detailed description of this crude model.

General theory along with thorough technical explanation of the Bayesian approach are described elsewhere [20]. Programs to take on the Bayesian approach were implemented in Matlab (The MathWorks, Inc. Natick, MA, USA) and the source code is available online in the Supplementary Materials. The Matlab codes are not generic, so the user will have to tweak them to fit his/her needs.

## 3. Results

For this analysis, we used $b_{d}=2.5/{\left(l_{d}-1\right)}^{2}$ for setting the roughness parameters $b_{d}$ in Equation (5), where $l_{d}$ represents the number of levels in dimension *d*. The rationale for this is that exp$\left\{-{\left(l_{d}-1\right)}^{2}b_{d}\right\}$ represents the correlation between the utility values for two health states differing only in that one is at level 1 and the other at level $l_{d}$ in dimension *d* [18]. This choice of $b_{d}$ makes this equal to exp(−2.5) = 0.08. Note that this value is arbitrary; however, it reflects a belief that the deviations of the true utility function from the linear additive form will not be dramatic.

The nonparametric Bayesian model (which we shall refer to as model (1)) was applied to estimate a Lebanon valuation, where the UK data were employed in the model as informative priors. The resulting estimates were then compared to those derived from modeling the Lebanon valuation alone (crude model).

**Figure 1:**
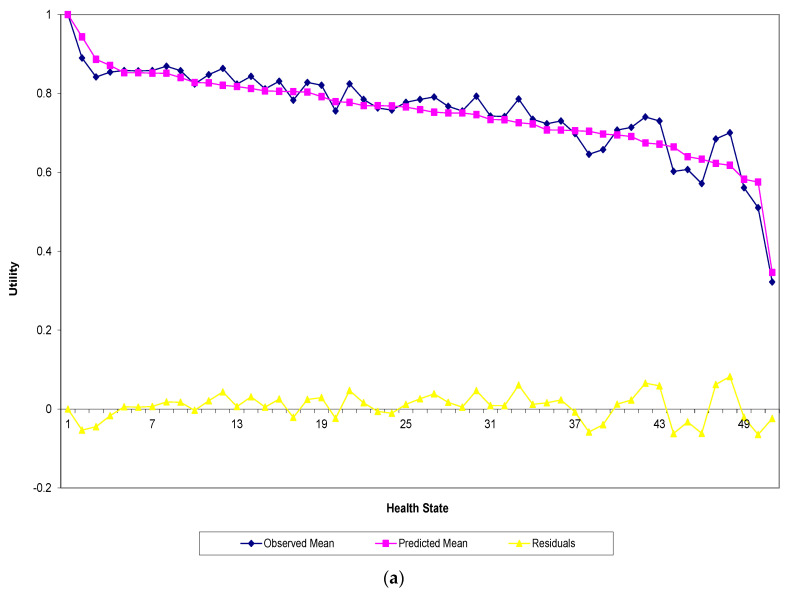

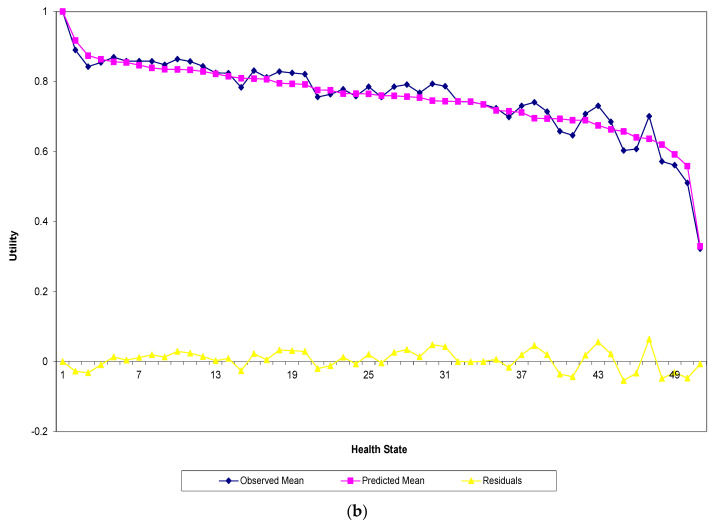
Actual and predicted mean health states valuations for (**a**) the crude model and (**b**) model (1).

The predictive ability of the two models are compared in Figure 1a,b, where the predicted (pink line) and observed (blue line) mean valuations for the 49 health states valued in the sample in addition to the full health were displayed, respectively, for both the crude model and model (1). In each figure, the errors are represented by the yellow line and were calculated by taking the difference between the two valuations. It is to be noted that the health states were sorted by the predicted mean valuations and plotted accordingly. When comparing the two figures, Figure 1a shows an obvious variation of the observed values around the predicted mean valuations obtained from the crude model, mainly for the moderate and poor health states values. In contrast, Figure 1b suggests that model (1) predicts the mean utilities quite well across the board.

**Figure 2:**
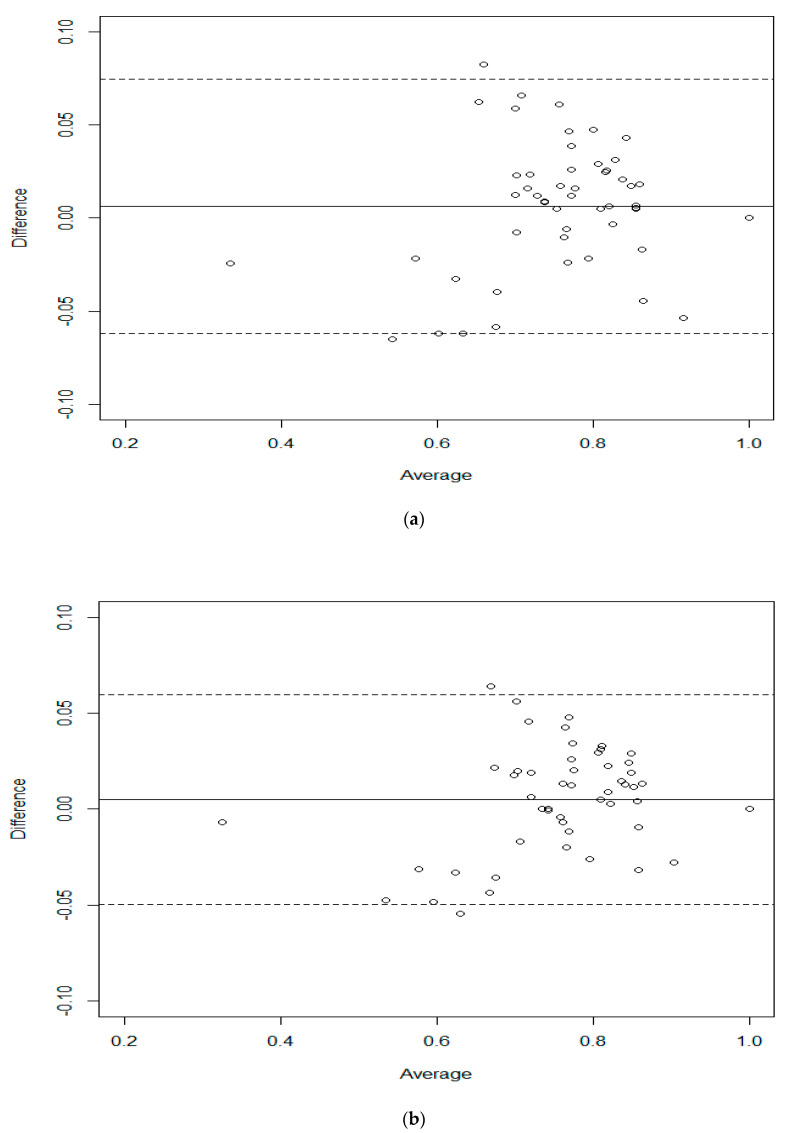
Bland–Altman plot for (**a**) the crude model and (**b**) model (1).

The Bland–Altman agreement plots [26] in Figure 2a (crude model) and Figure 2b (model (1)) indicate better and more precisely the quantification in terms of bias and/or precision. The difference between the predicted and actual mean utilities is displayed versus the mean bias in this context. The solid line in both figures indicates the mean bias, whereas the 95% limits of agreement were depicted by the dotted lines. When comparing the two plots, we can see that model (1) has greater agreement. This conclusion could be drawn from a variety of observations. First, the range of the 95% limits of agreement obtained from model (1) is 0.109, which is shorter than that of the crude model range of 0.136. Second, model (1) has a difference in mean bias of 0.0049, whereas the crude one scores a value of 0.0064. Finally, model (1) scores a difference standard deviation of 0.027, which is much smaller than that of the crude model with a value of 0.035.

The inferences for the utilities of the 49 SF-6D states in addition to the full health are displayed in Table 1. The observed mean utilities of the Lebanon data are displayed in column 2, whereas the UK predicted mean utilities and standard deviation that were employed as informative priors in model (1) are displayed in column 3 and 4, respectively. Additionally, the predicted mean utilities together with their standard deviations obtained from the crude model are displayed in column 5 and 6, respectively, whereas the corresponding estimates obtained from model (1) are displayed in column 7 and 8. It is evident from Table 1 that model (1) provides a better predictive performance in comparison to the crude overall, and as a result it scores a value of 0.028 for RMSE vs. 0.035 for the crude one.

Table 1 also shows another significant difference between the two models. For the pits state, for example, the crude model estimates a value of 0.3463, despite the fact that the observed mean for this health state is 0.3222, whilst the value from model (1) is 0.3292. There are also differences in the performance of the two models due to monotonicity (in which some good states are assigned a lower value than bad states). A total of 10,000 states were picked randomly with no replacement from a total of 18,000 states. It is perhaps worth noting that each of the 10,000 health states has 6–12 states adjacent to it, given they only vary in one dimension. Then, as a result of choosing one health state randomly from these 6 to 12 states, 10,000 adjacent pairs were generated. The results revealed that, out of these 10,000 adjacent pairs, 10% show non-monotonicity in model (1) in comparison to 20% for the crude one.

**Figure 3:**
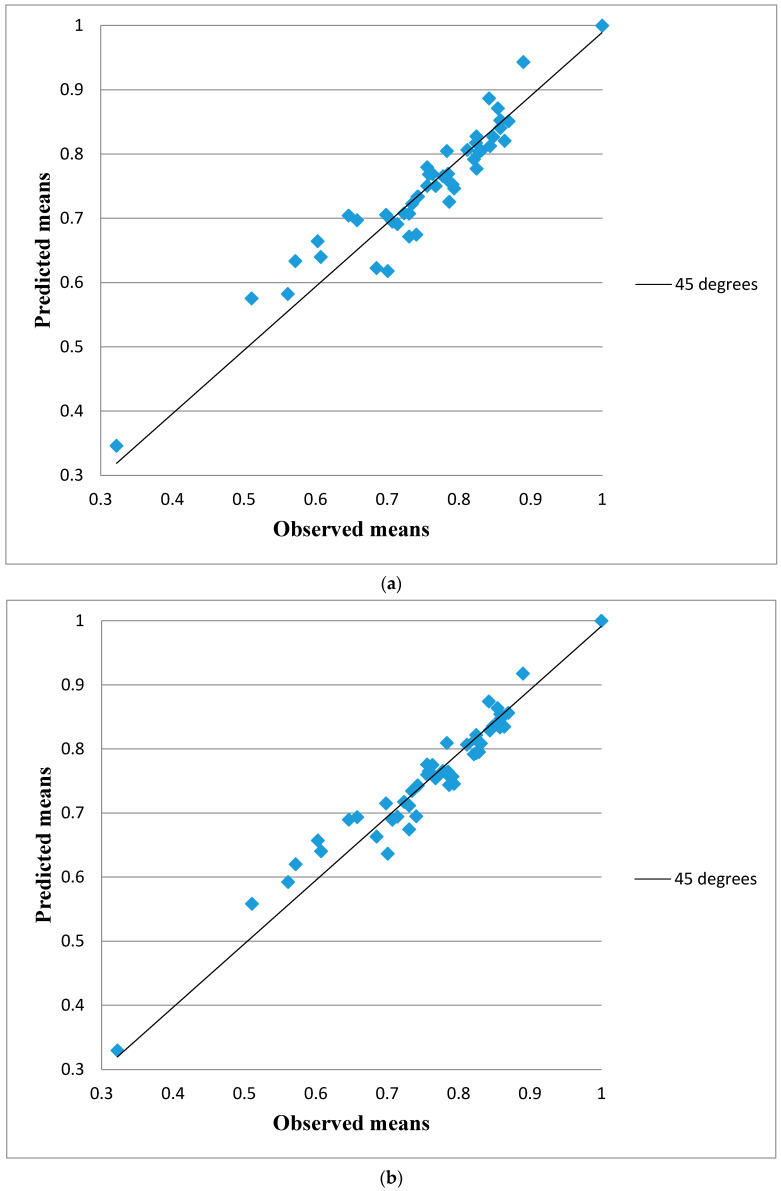
Actual and predicted utilities for (**a**) the crude model and (**b**) model (1).

Another aspect to show the difference in the performance between the two models is reflected in Figure 3, which displays the predicted mean utilities from the crude (Figure 3a) and model (1) (Figure 3b) versus the actual values of the 49 states, along with the 45° line of unity (solid line). In theory, the predicted mean utilities are expected to lie approximately on the unity line, hence this would be assessed as a good agreement. When comparing the two figures, we can see that model (1) has greater agreement, given the valuations tend to be closer to the perfect line, whilst the corresponding estimates obtained from the crude model tend to show a larger scatter as they deviate largely from the unity line. Therefore, we can stress that model (1) produces better predictions and is much more precise than the crude one.

To this end, it could be argued that it is hard to observe from the graphs and/or from the table a substantial difference in the performance of the two models (i.e., predictions) with respect to the data. However, the difference in terms of health and quality of life is substantial. Note that a key element in our study is the valuation of health states in order to calculate QALYs. Thus, extremely precise estimation of the health state utility values is an important component of this. As an example, note from Table 1 that health state 642,612 has a predictive utility value of 0.6228 from the crude model and 0.6633 from model (1), whereas the observed mean value for this health state is 0.685. Thus, the difference in utility estimates is almost 0.04. This could result in an increase in QALYs from a treatment that prolongs life by one year from 0.5 to 0.54, which for a treatment costing £10,000 would reduce the cost per QALY from £20,000 to £18,518 and make it below the cost effectiveness threshold used by the UK National Institute for Health and Clinical Excellence. That is, it could potentially influence whether or not a treatment or health care program is funded. This could, in turn, potentially impact on the validity of the resource allocation decisions made.

## 4. Discussion

In Kharroubi [20], a nonparametric Bayesian model was developed to allow results from one country to be used as prior information for a valuation study in a new country. In the present study, this model was applied to explore whether the use of Lebanon value sets alongside the existing evidence from the UK provides better valuation estimates than analyzing the Lebanon data alone. Analysis revealed that the UK evidence served as significant prior knowledge to the Lebanon analysis. More specifically, the model that includes the UK data as informative priors produced better predictions much more precisely than the model that excludes the UK data under all prediction criterion used, such as the estimated versus observed mean utilities, mean predicted error, and RMSE. This is a promising approach that suggests that already existing valuation studies can be merged with a smaller valuation study in another country to derive value sets, thereby making own country value sets more attainable for LMICs.

The approach provided in this paper is a replica of that used to model the US, Hong Kong, and Japan alongside existing UK data [20,21,22,23]. The previous analyses were based on the following assumptions: (1) cultural similarities between the nations under study, for example, the UK population’s preferences are very similar to those in the US; and (2) all countries in question had plenty of data. Typically, different countries might have different preferences, as well as different population compositions, work, cultures, and languages, all of which could potentially impact the relative values assigned to various health dimensions (for instance, physical functioning or vitality), and the position of every health state on the [0, 1] scale. As a result, the proposed approach may not always produce correct estimations. In the present study, we aimed to explore if such an approach could be used in countries with small populations and various demographic compositions, work, cultures, and languages, and if so, how generalizable these approaches may be by using experience from a European country, such as the UK, to facilitate the analysis of a value set in another Asian country or LMIC, such as Lebanon.

Experimental studies in different countries are needed for deriving value sets, such as the EQ-5D, or SF-6D. Typically, such work is very expensive and is potentially wasteful. The present work suggests that the use of the already existing data as potential prior information can improve the prediction accuracy. This offers the potential to reduce the number of states to be valued and so reduce the cost of cross-country valuation studies. This will be extremely crucial in countries without the same capacity to perform large-scale evaluation exercises, especially (1) in countries with small settings and LMICs or (2) in the context of data collection post the COVID-19 pandemic, through costly and time-consuming face-to-face interviews with techniques, such as SG and TTO.

It is perhaps noteworthy that our basic model (1) has the key benefit of allowing more than two countries to be analyzed. On this basis, Equations (3) and (4) may be generalized further to generic forms in order to handle *n* countries:

(7) $$E\left(u\left(x\right)\right)=\sum_{k=1}^{n}E\left(u_{k}\left(x\right)\right)+\gamma +\beta^{T}x$$

and variance–covariance matrix:

(8) $$\sum_{k=1}^{n}\mathrm{cov}\left(u_{k}\left(x\right),u_{k}\left(x^{\prime }\right)\right)+\sigma^{2}c\left(x,x’\right)$$

where $\overset{n}{\underset{k=1}{\sum }}E\left(u_{k}\left(x\right)\right)$ represents the total expected utility of state **x** and $\overset{n}{\underset{k=1}{\sum }}\mathrm{cov}\left(u_{k}\left(x\right),u_{k}\left(x^{\prime }\right)\right)$ represents the total variance-covariance matrix between $u_{k}\left(x\right)$ and $u_{k}\left(x^{\prime }\right)$ for two distinct states **x** and **x**′. Note that both $\overset{n}{\underset{k=1}{\sum }}E\left(u_{k}\left(x\right)\right)$ and $\overset{n}{\underset{k=1}{\sum }}\mathrm{cov}\left(u_{k}\left(x\right),u_{k}\left(x^{\prime }\right)\right)$ in Equations (7) and (8) are obtained from the analysis of the existing datasets in *n* different countries. Ongoing research is underway to demonstrate this idea for three different countries, namely the UK, HK, and Japan. In this respect, it is important to highlight that this also requires stronger assumptions: relevance of all included countries in the analysis, especially when data from one country are limited compared to those from another one. For example, if a large sample is available for the UK but only limited data are available from Japan, would it still be sensible to assign the same weights to the evidence estimated from these two countries when trying to improve the estimates for a country with only limited data available? It could be argued that this should be done in the context of the specific relevance that estimates from each country may have in terms of another one. In addition, prior sensitivity checks may become crucial to see how much giving different weights to the estimates of different countries may actually affect the results. All of the above are the subject of further work.

The analyses presented in this paper were carried out using empirical examples, which is beneficial and a valuable addition to the literature. Similar analysis via simulated data would be incredibly useful for further work in order to learn how valuations from different countries or ethnic groups vary, and to investigate the association between how different such countries are and how important the use of informative priors is. This in turn would allow for investigating the whole range of distances between national valuations [27]. Examining this would form a key research agenda for the future.

One limitation of the present work is that it is unclear whether a value set developed using an own country dataset modeled alongside a new country’s dataset tend to be acceptable, given various international funding agencies recommend the use of a country’s own valuations to calculate QALYs for use in CUA. However, when the estimations are correct or the ranking of health states and the position of each health state on the [0, 1] scale are similar to those derived through a large-scale evaluation exercise, this may not be a major concern. Another limitation is related to the quality of the data acquired in Lebanon and, in turn, the adequacy of our model formulation. The SF-6D valuation data from the pilot study in Lebanon should not be considered as a representative sample of the Lebanon general population, given the study sample was obtained from AUB where most of the respondents were highly educated. In the case when there is bad data with a poor signal to noise ratio, we believe this could bias the valuation results, suggesting that this approach may not always provide precise predictions. Future research with a more representative sample of the Lebanon general population is then encouraged to produce a Lebanese-specific SF-6D value set. However, because of the way that we modelled health state preference data using the nonparametric Bayesian method, it is unlikely that this would have an impact on the resulting estimates, though this could be assessed in further research.

## 5. Conclusions

The simple idea of using the UK results as informative priors to the Lebanon analysis proves to be significant in terms of generating better estimations than analyzing Lebanon data alone. Such an analysis (borrowing strength from existing countries’ valuations) tends to allow much smaller studies than have hitherto been used upon producing valuations for new countries. This kind of analysis will be hugely important in countries with a smaller setting and/or LMICs without the same capacity to conduct large-scale valuations, thereby making own country value sets more attainable. Ongoing research is underway to demonstrate this idea for more than two countries.

## Figures and Tables

**Table 1:** Posterior estimates for the 49 health states in addition to full health.

Health State	Observed Mean	UK		Crude Model		Model (1)	
		Posterior Mean	Posterior SD	Posterior Mean	Posterior SD	Posterior Mean	Posterior SD
111,111	1	1	0	1	0	1	0
111,621	0.8244	0.7482	0.0345	0.8276	0.0264	0.8218	0.0269
113,411	0.8544	0.7284	0.031	0.8713	0.0207	0.8637	0.022
115,653	0.7306	0.5652	0.0561	0.7073	0.0272	0.7116	0.0277
121,212	0.8422	0.8275	0.0261	0.8867	0.0194	0.8741	0.0209
122,233	0.8694	0.7475	0.034	0.8512	0.0235	0.8562	0.0259
122,425	0.7582	0.6784	0.0353	0.7686	0.0234	0.7652	0.0241
124,125	0.8478	0.7292	0.0475	0.8269	0.0245	0.835	0.0258
131,542	0.8283	0.6181	0.0304	0.8038	0.0236	0.7953	0.0257
132,524	0.7633	0.6574	0.037	0.7692	0.022	0.775	0.0232
133,132	0.8583	0.6942	0.0343	0.8517	0.0227	0.8467	0.0244
135,312	0.7556	0.6992	0.0488	0.7795	0.0221	0.7757	0.0229
142,154	0.7912	0.6844	0.0373	0.7527	0.0287	0.7569	0.0294
144,341	0.7418	0.72	0.0279	0.7335	0.0249	0.7425	0.0255
211,111	0.89	0.9197	0.0215	0.9434	0.0169	0.9176	0.0248
212,145	0.785	0.6927	0.0446	0.7693	0.0266	0.7591	0.0278
213,323	0.7833	0.7761	0.0296	0.8048	0.0208	0.8093	0.0215
221,452	0.8239	0.6237	0.0459	0.8178	0.0234	0.8148	0.0254
224,612	0.6461	0.6256	0.0392	0.7043	0.0238	0.6897	0.0244
232,111	0.8576	0.6987	0.0377	0.8525	0.0251	0.8336	0.0273
235,224	0.7676	0.6486	0.0335	0.7505	0.0241	0.7541	0.025
241,531	0.785	0.702	0.0352	0.7592	0.0251	0.7646	0.0266
312,332	0.8639	0.7472	0.0285	0.8207	0.0227	0.8348	0.0252
315,515	0.6983	0.6642	0.0363	0.7058	0.0247	0.7152	0.0247
321,122	0.8583	0.7638	0.0266	0.8527	0.0218	0.8542	0.0234
323,644	0.5717	0.5362	0.0287	0.6335	0.0256	0.6201	0.0257
332,411	0.8435	0.7217	0.0376	0.8125	0.0247	0.8289	0.0265
334,251	0.7347	0.6761	0.0532	0.7229	0.0251	0.7344	0.0258
341,123	0.8311	0.7009	0.0393	0.8055	0.0248	0.8085	0.0259
412,152	0.7933	0.6558	0.0371	0.7466	0.0251	0.7454	0.0277
414,522	0.7556	0.6612	0.0301	0.7505	0.023	0.7597	0.0235
421,314	0.8117	0.6689	0.0368	0.8067	0.0236	0.8066	0.025
425,131	0.6578	0.6771	0.0551	0.6973	0.0236	0.6935	0.0247
431,443	0.8247	0.638	0.0339	0.7775	0.027	0.7935	0.0274
432,621	0.7429	0.6468	0.0487	0.734	0.0237	0.7428	0.0254
443,215	0.7306	0.6548	0.0352	0.6718	0.0274	0.6746	0.0283
511,114	0.8578	0.6993	0.0379	0.8406	0.0247	0.8387	0.0267
512,242	0.6028	0.6906	0.0324	0.6647	0.0244	0.6573	0.0244
522,321	0.7778	0.6846	0.0324	0.7658	0.023	0.7654	0.0253
523,551	0.6072	0.6201	0.0471	0.6399	0.0257	0.6404	0.0261
531,635	0.7865	0.5323	0.0345	0.7258	0.0302	0.7438	0.0302
534,113	0.7235	0.7106	0.0437	0.7078	0.0249	0.7173	0.026
545,422	0.7006	0.6351	0.0322	0.6181	0.029	0.6365	0.0292
611,221	0.8211	0.6667	0.0521	0.7922	0.0253	0.7918	0.0275
614,434	0.5611	0.6497	0.0383	0.5826	0.0273	0.5922	0.0273
622,513	0.7072	0.5809	0.0392	0.6949	0.0259	0.6894	0.0275
625,141	0.5106	0.5561	0.0466	0.5755	0.0273	0.5582	0.028
631,355	0.7406	0.5823	0.0354	0.6749	0.0311	0.6948	0.0306
633,122	0.7141	0.6515	0.0338	0.6912	0.0249	0.6943	0.0261
642,612	0.685	0.5594	0.0336	0.6228	0.029	0.6633	0.0299
645,655	0.3222	0.3575	0.0186	0.3463	0.0252	0.3292	0.0237
RMSE				0.035		0.028	

## Data Availability

The data presented in this study are available on request from the corresponding author. The data are not publicly available due to privacy.

## Supplementary Materials

Supplementary Materials available at https://www.mdpi.com/article/10.3390/ijerph18168409/s1 online includes a software implementation in Matlab of the methods presented here and source code to reproduce the analysis.
